# Detecting red-lesions from retinal fundus images using unique morphological features

**DOI:** 10.1038/s41598-023-30459-5

**Published:** 2023-03-01

**Authors:** Maryam Monemian, Hossein Rabbani

**Affiliations:** Medical Image and Signal Processing Research Center, Isfahan University of Medical Sciences, Isfahan, Iran

**Keywords:** Biomedical engineering, Medical imaging

## Abstract

One of the most important retinal diseases is Diabetic Retinopathy (DR) which can lead to serious damage to vision if remains untreated. Red-lesions are from important demonstrations of DR helping its identification in early stages. The detection and verification of them is helpful in the evaluation of disease severity and progression. In this paper, a novel image processing method is proposed for extracting red-lesions from fundus images. The method works based on finding and extracting the unique morphological features of red-lesions. After quality improvement of images, a pixel-based verification is performed in the proposed method to find the ones which provide a significant intensity change in a curve-like neighborhood. In order to do so, a curve is considered around each pixel and the intensity changes around the curve boundary are considered. The pixels for which it is possible to find such curves in at least two directions are considered as parts of red-lesions. The simplicity of computations, the high accuracy of results, and no need to post-processing operations are the important characteristics of the proposed method endorsing its good performance.

## Introduction

One of the salient diseases of retina is Diabetic Retinopathy (DR) which is popular among the people with a long history of diabetes. DR is developed when the high blood sugar damages retinal vessels. This damage can be in term of making blocked or leaky blood vessels. At early stages, DR may not show any sign, however, it can even cause vision loss in the advanced stages. With respect to its epidemiological aspects, it should be noted that 16 million Americans are estimated to be infected with DR by 2050 and 3.4 million of them have vision-threatening complications. If DR is timely identified, it is possible to control its progressive trend. Therefore, the process of identification and monitoring of DR is of considerable importance^1,2^.

An appropriate tool for the evaluation of retinal health status is fundus imaging which provides a two dimensional representation from a three dimensional tissue^3–30^. Regarding the dangerous consequences of DR, fundus imaging is widely utilized for verifying retinal vasculature^31^. The most important manifestations of DR include red-lesions such as Micro-Aneurysms (MA) and Hemorrhages (HE) and bright lesions like cotton-wool regions and hard exudates. In order to identify DR and determine its stage, it is necessary to extract abnormalities from retinal images.

In the field of identifying DR signs and determining its severity, a number of research works have been performed. These works are explained in details in the next section.

In this paper, a new method for the extraction of red-lesions from retinal fundus images is suggested. This method includes a novel framework for detecting intensity changes in curved boundaries. After pre-processing which consists of contrast enhancement of retinal fundus images, a pixel-based verification is performed to determine whether or not the pixel of interest belongs to a red-lesion. The pixel-wise verification includes operations which find a circular neighborhood around each pixel. After finding the circular neighborhood, the pixels which are located near the region boundaries are verified from intensity point of view. The evaluation results show that the proposed method has an appropriate accuracy in finding the red-lesions with possibly simple computations.

Since DR is an important retinal disease having dangerous effects on retina, it is very important to monitor patients for DR signs such as red-lesions. In fact, to propose a new algorithm with possibly simple computations is of high importance.

The important contributions of the proposed method are as follows.

- To directly find the red-lesions without the need to detection or segmentation of optic disc, fovea or blood vessels.
- To model red-lesions with dark regions with approximately roundish boundaries.
- To propose a novel procedure for finding curved boundaries using simple computations without the need to complicated transforms such as curvelet
- Slight pre-processing operations at the start of algorithm execution which does not necessitate de-noising or color illumination processes.
- No need to prior training with a large number of images labeled with specialists.

The rest of paper is organized as follows. “Literature review” includes a review on the existing works in the related filed. In “Method”, the proposed method is explained in details. “Experimental results” presents the results of performance evaluation of the new method. Finally, the concluding remarks are described in “Conclusions”.

## Literature review

In this section, the most recent important works in the field of red-lesion detection in fundus images are reviewed.

In^3^ a red-lesion extraction method is proposed which firstly finds all the pixels located on the boundaries of red-lesions or blood vessels. Then, a framework is executed where a new parameter is computed for the windows around the pixel of interest. The feature discriminating the red-lesion from blood vessel is the lack of continuity in red-lesions for at least one direction.

In^4^ a method for the segmentation of red-lesions is proposed which firstly prepares the image via the omission of bright border, de-noising, color equalization, and contrast enhancement. After pre-processing, dark pixels are considered as candidates for red-lesions and they are grouped in super-pixels. The pixels with similar color and texture are located in one super-pixel and the neighboring super-pixels with the same color are combined. A graph-based framework is then utilized for segmenting the red-lesions.

In^5^ a method for detecting MAs is proposed. At first, pre-processing operations are performed which include illumination equalization, contrast enhancement, and smoothing. Peak detection procedure is used for extracting MAs since MAs are bright regions in pre-processed image. MA regions should contain at least one local maximum. For each candidate region, several features are extracted which are hessian matrix-based, shape and intensity features. Then, three classifiers like Adaboost, KNN and Naïve Bayes are used to classify the regions based on the extracted features.

In^6^ a method for the extraction of bright and dark lesions is suggested. After pre-processing operations, it is necessary to segment blood vessels and localize optic disc and fovea. Then, the image is decomposed into several layers to identify the candidate regions. Feature extraction is performed using fast correlation-based filter in the next step. Finally, a Multi-Layer Perceptron (MLP) is used to distinguish the true candidates.

In^7^ a method for grading DR and also lesion detection is proposed. The method consists of a fully convolutional network which uses a deep residual network (Res-Net) as its convolutional model. In^32^ a method based on deep learning is suggested which identifies MA and exudates. This is a patch-based method which verifies all patches with overlap from the existence of abnormality viewpoint. Then, a CNN-based approach classifies each patch into five classes including normal, MA, HM, exudate, and high-risk lesion. A deep learning method for the classification of retinal images into no-DR, mild, moderate, severe and proliferative DR is proposed in^33^. Pre-processing should be performed for the input image which consists of contrast enhancement, noise removal, cropping, color normalization and data augmentation. The main method contains two deep models the first of which is a CNN receives an image and classifies into one of the mentioned cases. The second one is a YOLOv3 model which localizes the DR lesions. Another deep learning approach for categorizing images into no-DR and DR cases is presented in^34^. The method firstly pre-processes the input image and segments it using the eigenvalues of Hessian matrix. Then, it employs a CNN to classify the images using their segmented versions. In^35^ a lesion localization model based on deep learning approach is proposed which works on a patch-based procedure. The image is divided to square patches and the model contains two CNNs for selecting training patches. In the training phase each patch is labeled as lesion or no-lesion. The second CNN is trained with more challenging patches which are misclassified by the first CNN. In order to detect red-lesions in the retinal fundus images, a method is proposed in^9^ where deep learned features and handcrafted features are used. With respect to deep-learned features, a light convolutional neural network is utilized. As well as deep features, several handcraft features are also used for training a random forest classifier.

A MA detection method is presented in^8^. At first blood vessels are segmented with the analysis of eigenvalues of Hessian matrix. Then, a patch-based approach is designed to determine whether or not each patch includes MA. The discriminating features for each patch are extracted with the attention to local contrast in several directions.

A method for detecting all retinal lesions is proposed in^10^. Firstly, blood vessels and optic disc are extracted to reduce the false positives for dark and light lesions, respectively. Then, pre-processing operations including edge enhancement by curve-let transform are performed. In order to detect candidate lesions, Laplacian of Gaussian (LoG) filter and matched filter are utilized.

In this paper, a novel method is proposed for detecting red-lesions using the shape characteristics of lesions. Since the shape of red-lesions can be approximated with regions with roundish boundaries, the proposed method looks for the roundish boundaries in the fundus image. In order to do so, a pixel-wise approach is performed to determine whether or not the neighbor regions of each pixel can be part of a red-lesion. The method is capable of providing good accuracy with simple computations and without the need to detection or segmentation of blood vessels and optic disc. Also, there is no need to illumination correction or transforming the input image to other color spaces in the initialization step.

## Method

In this section, the new method called Circular Neighborhood Analysis for Red-lesion Detection (CNARD) is explained in details. The method consists of two main phases which are pre-processing and pixel-wise verification for detecting red-lesions. All phases are detailed in the following sections.

### Pre-processing

In order to prepare the image for processing operations in the next phase, it is necessary to improve the image quality in this phase. A retinal fundus image consists of three red, green and blue channels. The fundus image in the green channel has the highest contrast in comparison to other channels. Thus, the green channel is extracted and utilized for more processing. In order to improve the contrast of the image in the green channel, Contrast Limited Adaptive Histogram Equalization (CLAHE) is applied.

### Pixel-wise verification

In this phase, the required operations for detecting red-lesions are explained. Before explaining them, the main idea of CNARD for detecting red-lesions is described here. A red-lesion is a comparatively dark area providing intensity changes in several directions. Also, red-lesions can be considered as the regions with approximately roundish boundaries. In fact, the intensity changes occur near the roundish boundaries of red-lesions. Therefore, the identification of roundish boundaries around all the pixels of interest is the first step for identifying the red-lesions. Then, the pixels near those boundaries are verified from intensity viewpoint and the red-lesions are extracted.

Let *I* denote a retinal fundus image with *m* rows and *n* columns. Let *p*_*i,j*_ denote the pixel located in *i*th row and *j*th column.

**Figure 1:**
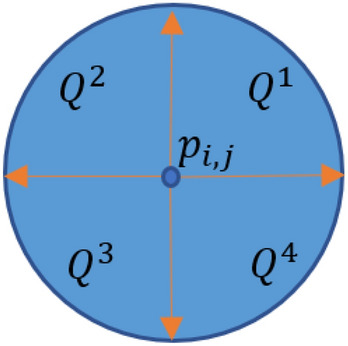
Different circle quarters centered at *p*_*i,j*_.

For each pixel, a full circle with* a*_*0*_ radius is considered. The center of circle is the pixel of interest. Then, four circle quarters located at the top-right, top-left, bottom-right and bottom-left parts of the mentioned circle are verified. Let ${Q}^{k}$ (*k* = 1, 2, 3, 4) denote the *k*^th^ circle quarter. Figure 1 presents all *Q*^*k*^s centered at *p*_*i,j*_.

We have a set of conditions which should be verified for all four circle quarters. In fact, if the conditions are true for a circle quarter, the related quarter can be considered as a candidate region for a red-lesion. In the following, the conditions verified for each circle quarter are explained.

### Conditions

In each of the mentioned quarter circles, different angles are considered from the center pixel. Using these different angles, it is possible for us to find the pixels located near the roundish boundary. Let $\theta$ denote the angel in the range of [0,${\theta }_{0}$]. We have considered seven values for $\theta$ which are 0, $\frac{\pi }{12}$, $\frac{\pi }{6}$, $\frac{\pi }{4}$, $\frac{\pi }{3}$,$\frac{5\pi }{12}$ , $\frac{\pi }{2}$ and uniformly distributed in [0,$\frac{\pi }{2}$]. For each value of $\theta$, we find the pixels which are located near the roundish boundary and we call them the corresponding points across $\theta$. Then, we compute the difference between intensity values of the corresponding points across the same angel. If for the majority of $\theta$ values in the related quarter, the absolute value of difference is sufficiently high and the farther points are lighter than the closer ones, the related quarter centered at *p*_*i,j*_ is considered as a candidate region for a red-lesion.

**Figure 2:**
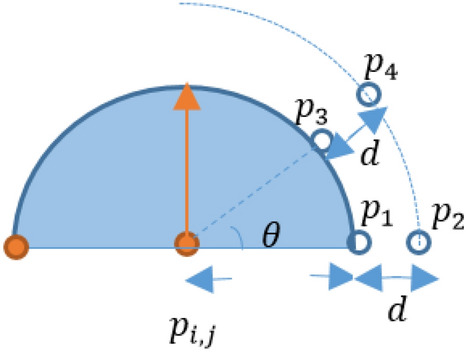
Example of a circle quarter and corresponding points along two certain angles.

For simplicity, we present a picture to show corresponding points for two different values of ϴ. In the following figure (Fig. 2), *p*_*1*_ and *p*_*2*_ are the corresponding points in the direction of $\theta =0$. Also, *p*_*3*_ and *p*_*4*_ are the corresponding points in the direction of $\theta =\frac{\pi }{4}$. In the proposed method, the difference between intensity values of the corresponding points in different directions is computed. For instance, the difference between intensity values of *p*_*3*_ and *p*_*4*_ is computed. It is necessary to have a sufficiently high difference value for corresponding points across different angles in a circle quarter. Also, the farther points should be lighter than the closer ones in the corresponding points. If these conditions are true for the majority of values of $\theta$ in a quarter, the quarter can be considered as a candidate for red-lesion.

**Figure 3:**
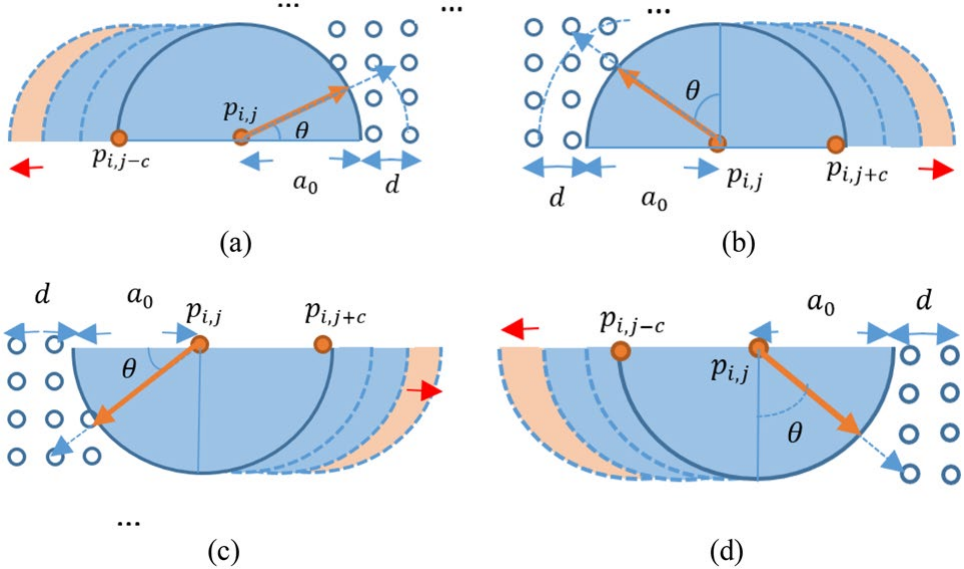
A schematic for *p*_*i,j*_ and its roundish neighborhood in (**a**) top-right and (**b**) top-left, (**c**) bottom-left and (**d**) bottom-right parts.

In order to clarify the required notations, several schematics are presented for all circle quarters in parts a, b, c, and d of Fig. 3, respectively.

For more clarification, we explain a sample case in part (a) of Fig. 3. If we move across the orange line, we can find the pixel located on the roundish boundary with radius *a*_*0*_. Such a pixel denoted by ${p}_{0}^{\theta ,1}$ has a horizontal component ${x}_{0}^{\theta ,1}$ and a vertical component ${y}_{0}^{\theta ,1}$ in comparison to *p*_*i,j*_. The values of ${x}_{0}^{\theta ,1}$ and ${y}_{0}^{\theta ,1}$ are equal to [${a}_{0}cos(\theta )$] and $[{a}_{0}sin(\theta )]$, respectively. The actual location of ${p}_{0}^{\theta ,1}$ is in ${\left(i-{y}_{0}^{\theta ,1}\right)\mathrm{th}}$ row and ${(j+{x}_{0}^{\theta ,1})\mathrm{th}}$ column. In fact, ${p}_{0}^{\theta ,1}$ is ${p}_{i-{y}_{0}^{\theta ,1},j+{x}_{0}^{\theta ,1}}$. Also, we continue until the radius of ${a}_{0}+d$ to find the next pixel in the same direction. This pixel denoted by ${p}_{1}^{\theta ,1}$ has a horizontal component ${x}_{1}^{\theta ,1}$ which is equal to [$({a}_{0}+d)cos(\theta )$] and a vertical component ${y}_{1}^{\theta ,1}$ equal to $[({a}_{0}+d)sin(\theta )]$. After finding the mentioned pixels, we compute the difference between their intensity values. Let *X*(*p*_*i,j*_) denote the intensity of *p*_*i,j*_. If ${p}_{1}^{\theta ,1}$ is lighter than ${p}_{0}^{\theta ,1}$ and the absolute difference between their intensity values is higher than a threshold denoted by *diff_th*, $\theta$ is considered as an angle satisfying the condition. Let $e\theta$ denote the value added to $\theta$ in each round. Also, we define a threshold denoted by *number_th*. The number of $\theta$ values for which the mentioned conditions are true should be higher than *number_th*.

It should be mentioned that the same procedure is executed in other circle quarters. Let ${p}_{0}^{\theta ,k}(k=\mathrm{2,3},\mathrm{4,0}\le \theta \le \frac{\pi }{2})$ denote the pixel located at the radius of ${a}_{0}$ and angle of $\theta$ from *p*_*i,j*_ in the *k*th circle quarters. Also, let ${p}_{1}^{\theta ,k}(k=\mathrm{2,3},\mathrm{4,0}\le \theta \le \frac{\pi }{2})$ denote the pixel located at the radius of $({a}_{0}+d)$ and angle of $\theta$ from *p*_*i,j*_ in the *k*th circle quarters. The exact locations of these points in different circle quarters are defined in the following equations.

For the second circle quarter we have,

1 $${p}_{0}^{\theta ,2}={p}_{i-{y}_{0}^{\theta ,2},j-{x}_{0}^{\theta ,2}}$$

2 $${p}_{1}^{\theta ,2}={p}_{i-{y}_{1}^{\theta ,2},j-{x}_{1}^{\theta ,2}}$$

where in (1) and (2) ${x}_{0}^{\theta ,2}=[{a}_{0}sin(\theta )]$, ${y}_{0}^{\theta ,2}=[{a}_{0}cos(\theta )]$, ${x}_{1}^{\theta ,2}=[({a}_{0}+d)sin(\theta )]$, and ${y}_{1}^{\theta ,2}=[({a}_{0}+d)cos(\theta )]$.

For the third circle quarter we have,

3 $${p}_{0}^{\theta ,3}={p}_{i+{y}_{0}^{\theta ,3},j-{x}_{0}^{\theta ,3}}$$

4 $${p}_{1}^{\theta ,3}={p}_{i+{y}_{1}^{\theta ,3},j-{x}_{1}^{\theta ,3}}$$

where in Eqs. (3) and (4) ${x}_{0}^{\theta ,3}=[{a}_{0}cos(\theta )]$, ${y}_{0}^{\theta ,3}=[{a}_{0}sin(\theta )]$, ${x}_{1}^{\theta ,3}=[({a}_{0}+d)cos(\theta )]$, and ${y}_{1}^{\theta ,3}=[({a}_{0}+d)sin(\theta )]$.

Finally, for the fourth circle quarter we have,

5 $${p}_{0}^{\theta ,4}={p}_{i+{y}_{0}^{\theta ,4},j+{x}_{0}^{\theta ,4}}$$

6 $${p}_{1}^{\theta ,4}={p}_{i+{y}_{1}^{\theta ,4},j+{x}_{1}^{\theta ,4}}$$

where in Eqs. (5) and (6) ${x}_{0}^{\theta ,4}=[{a}_{0}sin(\theta )]$, ${y}_{0}^{\theta ,4}=[{a}_{0}cos(\theta )]$, ${x}_{1}^{\theta ,4}=[({a}_{0}+d)sin(\theta )]$, and ${y}_{1}^{\theta ,4}=[({a}_{0}+d)cos(\theta )]$.

For each quarter circle (*k* = 1, 2, 3, 4), we compute the difference between intensity value of ${p}_{0}^{\theta ,k}$ and ${p}_{1}^{\theta ,k}$. If the absolute value of such a difference is higher than *diff_th* and ${p}_{1}^{\theta ,k}$ is lighter than ${p}_{0}^{\theta ,k}$, there is a significant intensity change in the direction of $\theta$. If the number of values of $\theta$ in a circle quarter with such conditions are greater than *number_th,* the related quarter is considered as a candidate for red-lesion.

**Figure 4:**
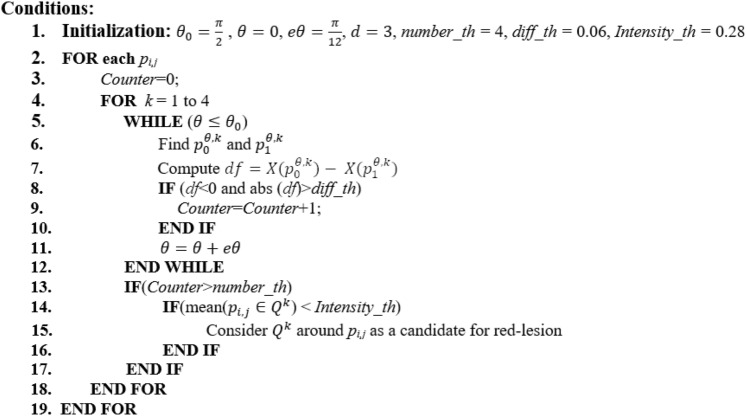
Pseudo-code for pixel-wise verification.

The pseudo-code for the CNARD method in the phase of pixel-wise verification is presented in Fig. 4.

In the pseudo-code of Fig. 4, it is not necessary to verify all conditions of all circle quarters in lines 4 to 18. In fact, if the top-right (top-left) circle quarter centered at a pixel of interest satisfies the conditions, we do not verify the conditions for bottom-right (bottom-left) circle quarter centered at that pixel.

After finding candidate circle quarters, it is necessary to find the real red-lesions and remove false positives. In order to do so, we search and find a *Q*^2^ circle quarter satisfying the conditions, in the left side of a *Q*^1^ circle quarter that have the conditions. In addition, we should find a *Q*^1^ circle quarter having the conditions, in the right side of a *Q*^2^ circle quarter which satisfies the conditions. If it is possible to find such circle quarters, the regions located between the mentioned *Q*^1^ and *Q*^2^ circle quarters, are considered as red-lesions. However, if it is not possible to find a *Q*^2^ circle quarter (*Q*^1^ circle quarter) in the left (right) side of a *Q*^1^ circle quarter (*Q*^2^ circle quarter), no red-lesion is identified.

It should be noted that it may be possible to have a pixel (*p*_*i,j*_) for which only one circle quarter (*Q*^1^, *Q*^2^, *Q*^3^, or *Q*^4^) is a candidate region for red-lesion. In such conditions, we can have two cases. In the first case, *Q*^1^ (*Q*^4^) quarter satisfies the conditions and it is a candidate for a red-lesion. We verify *Q*^2^ (*Q*^3^) quarter centered at *p*_*i,j*_ and we observe that they do not satisfy the conditions. If *Q*^2^ (*Q*^3^) quarters centered at *p*_*i,j*_ is a dark region, we can also continue the search process in the left side. We continue the search process in the left side to find a pixel for which *Q*^2^ (*Q*^3^) quarter satisfies the conditions. The search process stops if a pixel with such conditions is found or *Q*^2^ (*Q*^3^) quarter is light.

Parts a and d of Fig. 3 present the first case. Suppose that there is a pixel denoted by *p*_*i,j-c*_ in the left side of *p*_*i,j*_ for which *Q*^2^ (*Q*^3^) quarter satisfies the conditions. As can be observed in the figure, a red arrow can be observed under (above) this region. In such conditions all the square patches between *p*_*i,j*_ and *p*_*i,j-c*_ plus the *Q*^1^ (*Q*^4^) quarter centered at *p*_*i,j*_ and the *Q*^2^ (*Q*^3^) quarter centered at *p*_*i,j-c*_ are considered as red-lesions.

In the second case, the *Q*^2^ (*Q*^3^) quarter centered at *p*_*i,j*_ has the conditions and it is a candidate for a red-lesion. We verify the *Q*^1^ (*Q*^4^) quarter and we observe that the conditions are not true for it. If the *Q*^1^ (*Q*^4^) quarter centered at *p*_*i,j*_ is a dark region, we can also continue the search process in the right side. We continue the search process in the right side to find a pixel for which *Q*^1^*(Q*^4^) quarter has the conditions. The search process stops if a pixel with such conditions is found or we find a light quarter at the top-right (bottom-right) side of a pixel.

Parts b and c of Fig. 3 present this case. Suppose that there is a pixel denoted by *p*_*i,j*+*c*_ in the right side of *p*_*i,j*_ for which *Q*^1^ (*Q*^4^) satisfies the conditions.

As can be observed in the figure, a red arrow can be observed under (above) this region. In such conditions all the square patches between *p*_*i,j*_ and *p*_*i,j*+*c*_ plus the *Q*^1^ (*Q*^4^) quarter centered at *p*_*i,j*+*c*_ and the *Q*^2^ (*Q*^3^) quarter centered at *p*_*i,j*_ are considered as red-lesions.

## Experimental results

In this section, the results obtained from the process of performance evaluation are presented. The datasets we have utilized for testing the performance of the proposed method are DIARETDB1^36^, DIARETDB0^37^ and Kaggle^38^ which all of them are public. DIARETDB1 consists of 89 fundus images where 84 images of them have at least a sign of DR and five images are normal. Also, DIARETDB0 includes 130 fundus images where 20 images are normal and 110 images consists of DR abnormalities.

In order to evaluate the performance of CNARD, a program has been run in MATLAB 2018b. The performance metrics utilized for evaluating the proposed method are sensitivity and specificity. Sensitivity denoted by *SE* is defined as the number of true positives (*TP*) to the sum of *TP*s and false negatives (*FN*). Furthermore, specificity denoted by *SP* is defined as the number of true negatives (*TN*) to the sum of *TN*s and false positives (*FP*). Moreover, accuracy which is denoted by *ACC* is defined as the ratio of sum of *TP*s and *TN*s to the sum of *TP*s, *TN*s, *FP*s, and *FN*s. In order to measure the intersection between the extracted parts and the ground truth, we consider Intersection Over Opinion (IOO). IOO is defined as follows.

7 $$IOO=\frac{Area\, of\, intersection}{Ground\, Truth\, Area+Predicted\, Region\, Area-Area\, of\, intersection}$$

**Figure 5:**
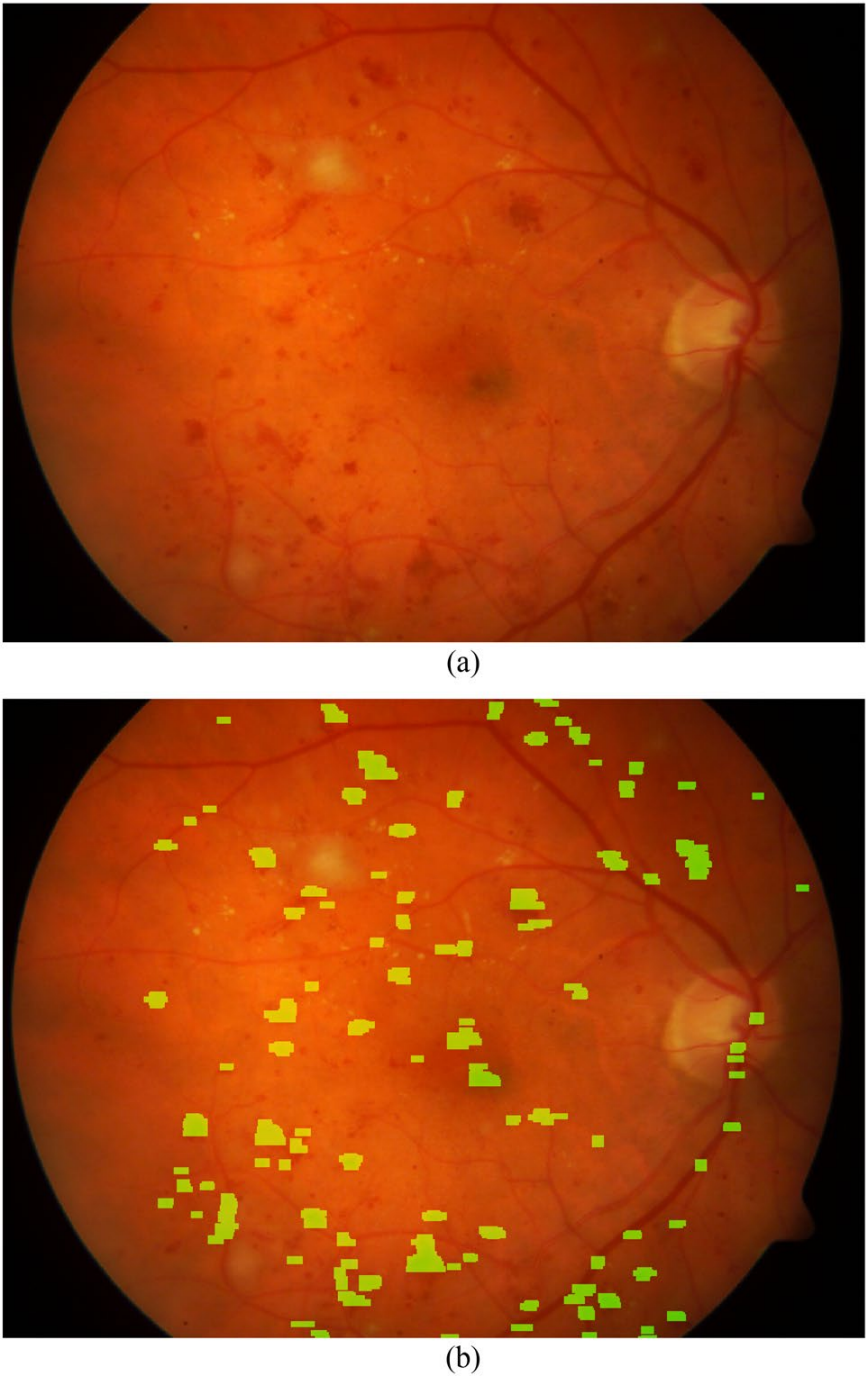
(**a**) A retinal fundus image, (**b**) the extracted red-lesions.

We also consider a value of 0.8 for IOO threshold. In fact, if the value of IOO for an extracted area is 0.8, the area is considered to be a TP. The values of parameters used for simulating the proposed method are presented in Table 1. Figure 5 presents a fundus image and its red-lesions extracted by CNARD method. As can be observed, the existing red-lesions are successfully extracted by CNARD method.

**Table 1:** The values of parameters utilized for simulation.

Parameter	Value
*a*_0_	10
*D*	3
*number_th*	4
*diff_th*	0.06
*Intensity_th*	0.28
$e\theta$	$\frac{\pi }{12}$

**Figure 6:**
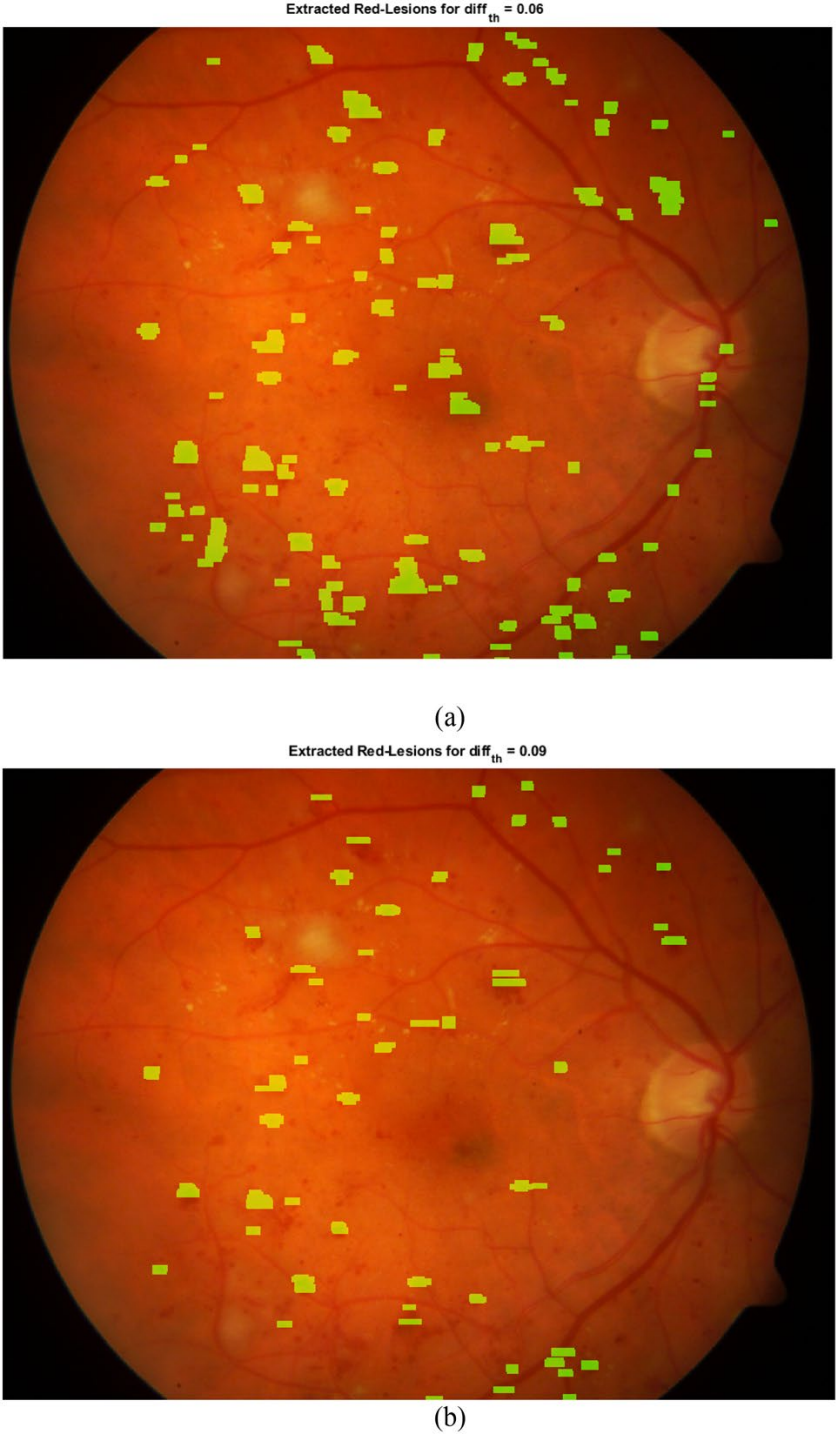
The effect of two values of *diff_th* on the capability of red-lesion detection.

In addition, the value of parameters affects the sensitivity and specificity of the proposed method. For instance, if the value of *diff_th* increases, the conditions for identifying a red-lesion become more difficult. Therefore, it may be possible to miss some correct red-lesions. Also, in such conditions, it may be possible to reduce the number of false positives. Figure 6 presents the extracted red-lesions for two different values of *diff_th*.

**Figure 7:**
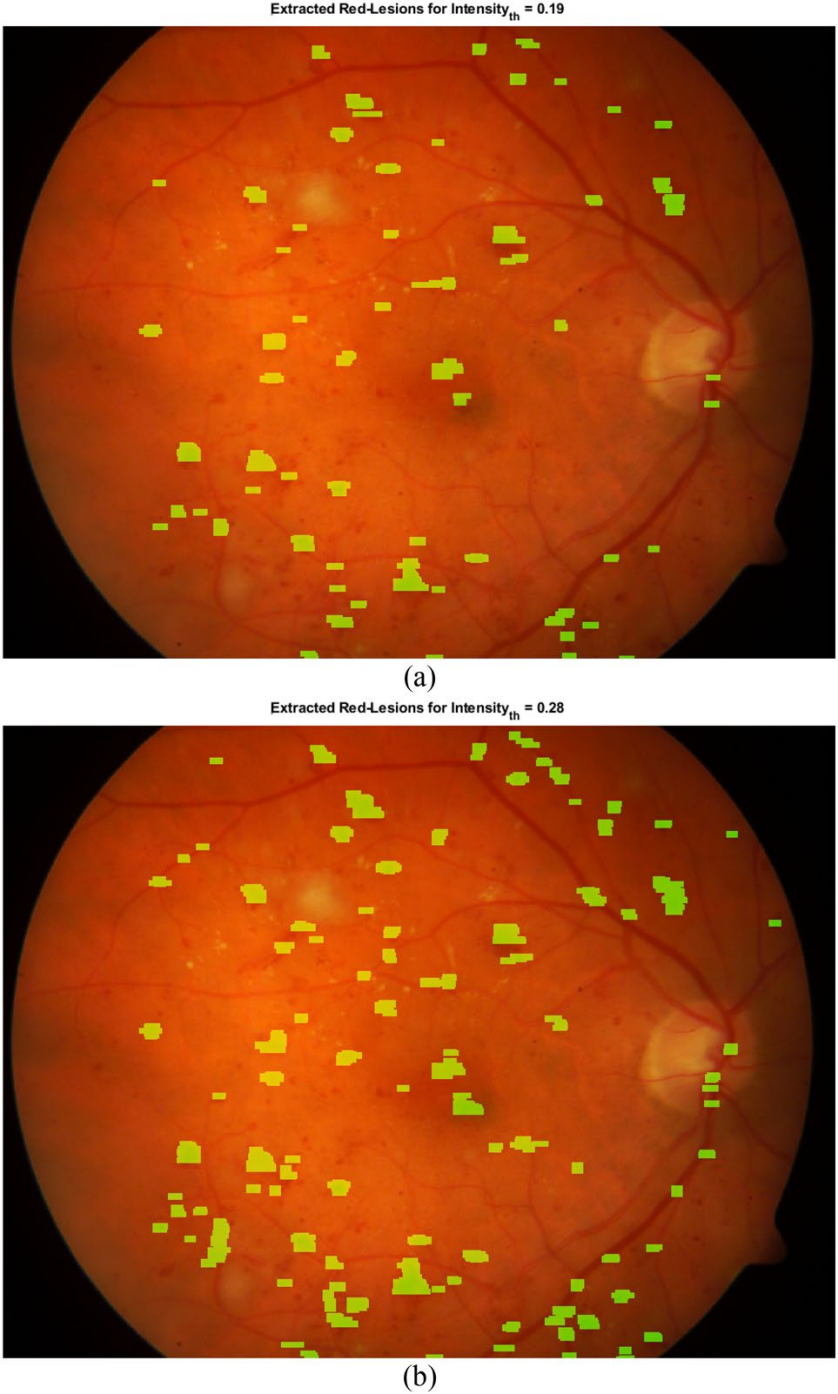
The effect of two values of *Intensity_th* on the capability of red-lesion detection.

Furthermore, the value of *Intensity_th* affects the sensitivity and specificity of CNARD method. Figure 7 presents the extracted red-lesions for different values of *Intensity_th*. As can be observed, for the smaller value of *Intensity_th*, the conditions for considering a region as a right-region or left-region for red-lesion become more difficult. Thus, for the smaller value of *Intensity_th*, some correct red-lesions may be missed.

Table 2 presents the results of CNARD method and also three other methods for detecting red-lesions. The research works chosen for comparison are^3,4^. In^3^ a red-lesion extraction method is proposed which firstly find the pixels located on the boundaries of dark regions like a red-lesion or blood vessel. Then, a discriminating feature is extracted to separate the pixels located on red-lesion boundaries from others. This feature is extracted by considering neighbor windows around the boundary pixels in several directions.

**Table 2:** Sensitivity, specificity and accuracy values for CNARD method, REDICA^3^, the methods of^4,39–41^ for (a) DIARETDB0 and (b) DIARETDB1, (c) Kaggle.

(a)						
Parameter	CNARD	REDICA^3^				
*SE*	0.92	0.89				
*SP*	0.95	0.9				
*ACC*	0.95					

(b)						
Parameter	CNARD	REDICA^3^	^4^	^39^	^40^	^41^
*SE*	0.93	0.87	0.84	0.83	0.83	0.76
*SP*	0.96	0.88	0.88		0.97	0.76
*ACC*	0.96					0.76

(c)						
Parameter	CNARD	REDICA^3^				
*SE*	0.88	0.82				
*SP*	0.95	0.9				
*ACC*	0.94					

In^4^ the segmentation of red-lesions is performed in several steps. In the first step, the quality of image is improved through de-noising, color equalization, the omission of bright border, and contrast enhancement. After pre-processing, dark pixels are considered as candidates for red-lesions and the super-pixels are formed by grouping them. A super-pixel is formed by the pixels with similar color and texture. In order to segment the red-lesions, a graph based framework is utilized.

In^39^ a method for automatic detection of red-lesions is proposed where a CNN model identifies the targets. The CNN model is Darknet53 including 106 layers for residual networks, skip connection and up-sampling. The output is produced by applying proper kernels on the feature maps of different sizes. The model also utilizes squared error loss for the penalizing error loss. The sensitivity result of^39^ is presented in Table 2. As it is obvious in the table, the sensitivity of our proposed method for DiaretDB1 is 93% which is considerably higher than 83% of^39^.

A method for detecting red-lesions is suggested in^40^ which works based on the analysis of super-pixels. Super-pixels contain the less redundancy and complexity compared to pixel-based approaches. In this method, candidates are firstly extracted from the retinal fundus image. Then, a large number of features in multi-channel images are extracted from the input image. Finally, Fisher Discriminant Analysis (FDA) is utilized for the classification purpose. However, it is also necessary to perform post-processing step in terms of fovea and blood vessel detection. As it is clear in Table 2, the sensitivity value of^40^ is 0.83 which is highly smaller than the proposed method. Our specificity value is 0.96 which is slightly smaller than 0.97 of^40^ method. The value of ACC for^3,4,39,40^ has not been reported in the mentioned references. Also, the value of Average IOO in our proposed method is equal to 0.84, 0.85 and 0.83 in Diaretdb0, Diaretdb1, and Kaggle datasets, respectively.

In^41^, a method is proposed where the abnormal pathologies of retinal fundus images are categorized. In this method, the whole fundus image is divided into patches and a series of features are extracted for each patch based on the texture and morphology of the patch. With respect to texture feature, rotation-invariant Local Binary Pattern (LBP) is utilized which is a type of LBP texture descriptors. The morphological features are extracted using granulometry technique. Then, several different classifiers including Random Forests (RF), Gaussian processes and SVM utilizes the extracted features to categorize the abnormal pathologies. As can be observed in the table, the values of sensitivity, specificity and accuracy in the proposed method is considerably higher than^41^.

In the proposed method, there is no need to save special information. In fact, it is sufficient to save the intensity values of image pixels. However, in the method of^3^ it is necessary to find the boundary points in several directions and save the status of each pixel to determine whether or not it is boundary. In fact, it is necessary to define several matrices for each direction. In each matrix, the status of each pixel in each direction is determined through the processing operations in the second phase of the method. Therefore, the memory requirement in the method of^3^ is higher than the proposed method. With respect to execution time and computational complexity, the execution time of the proposed method is less than the method of^3^. The reason is the necessity of finding all boundary points and verifying the necessary conditions and optic disc removal in the post-processing in the method of^3^.

## Conclusions

In this paper, a new method is proposed for detecting red-lesions which are from important symptoms of DR. The fact utilized by the proposed method is that red-lesions have approximately roundish boundaries. Therefore, the roundish boundaries at which significant intensity changes are observed should be identified. The method consists of two phases which are pre-processing and pixel-wise verification. In the first phase, the quality of image is improved to prepare it for more processing. In the next phase, a pixel-wise approach is designed where for each pixel a full circle centered at the pixel of interest is considered. A set of conditions are defined to verify the right and left quarter circles. For each circle quarter, several angles are considered to move across and find the pixels located on certain radius values. If for the majority of angles, there is a high difference with special sign between the corresponding points, the related part is considered as a candidate part for a red-lesion. For each circle quarter satisfying the conditions in the right (left) side, it is necessary to find a circle quarter which satisfies the conditions in the left (right) side to make sure they form a red-lesion. The method has been executed on two public datasets and the experimental results show its good performance in comparison to the existing methods. In the future, this research work can be extended to the extraction of bright lesions or classifying different types of red-lesions using textural features.

## Data Availability

The datasets generated and/or analyzed during the current study are available in the following links. https://www.it.lut.fi/project/imageret/diaretdb1/, https://www.it.lut.fi/project/imageret/diaretdb0/, https://www.kaggle.com/c/diabetic-retinopathy-detection.
